# Cost-effectiveness of quality improvement intervention to reduce time between CT-detection and ureteroscopic laser fragmentation in acute symptomatic ureteric stones management

**DOI:** 10.1007/s00345-023-04694-4

**Published:** 2024-03-13

**Authors:** Faid Khopekar, Soha Nabi, Mehdi Shiva, Morven Stewart, Benedict Rajendran, Ghulam Nabi

**Affiliations:** 1 Ninewells Hospital, Dundee, UK; 2 University of Aberdeen, Aberdeen, UK; 3 University of Oxford, Oxford, UK; 4 Division of Imaging Sciences and Technology, School of Medicine, Ninewells Hospital, Dundee, DD1 9SY UK

**Keywords:** Ureteroscopy, Laser, Stone fragmentation, Stent, Cost effectiveness

## Abstract

**Objective:**

To prospectively assess clinical and cost effectiveness of emergency ureteroscopic laser fragmentation of urinary stones causing symptoms or obstruction**.**

**Patients and methods:**

100 consecutive patients with an average (median) age 55.6 (57.5) years and average (median) stone size of 8.2 mm (± 7 mm) between October 2018 and December 2021 who underwent emergency ureteroscopy and laser fragmentation formed the study cohort as part of a clinical service quality improvement. Primary outcome was single procedure stone-free rate and cost-effectiveness. The secondary outcomes were complications, re-admission and re-intervention. A decision analysis model was constructed to compare the cost-effectiveness of emergency ureteroscopy with laser fragmentation (EUL) and emergency temporary stenting followed by delayed ureteroscopy with laser fragmentation (DUL) using our results and success rates for modelling.

**Results:**

Single procedure stone-free rates (SFR) for EUL and DUL were 85%. The re-intervention rate, re-admission and complication rates of the study cohort (EUL) were 9%, 18%, and 4%, respectively, compared to 15%, 20%, and 5%, respectively for the control cohort (DUL). The decision analysis modelling demonstrated that the EUL treatment option was more cost-efficient, averting £2868 (€3260) per patient for the UK health sector. Total cost of delayed intervention was £7783 (€8847) for DUL in contrast to £4915 (€5580) for EUL.

**Conclusions:**

Implementation of quality improvement project based on a reduction in CT detection-to-laser fragmentation time interval in acute ureteric obstruction or symptoms caused by stones had similar clinical effectiveness compared to delayed ureteroscopic management, but more cost-effective.

**Supplementary Information:**

The online version contains supplementary material available at 10.1007/s00345-023-04694-4.

## Introduction

Studies show that there has been a significant increase in the prevalence of urological stone disease in the UK and around the globe over the last decade [1, 2]. According to data extracted from the Hospital Episode Statistics website [3], there has been a 63% increase in the incidences of urinary tract stones between 2000 and 2010. Since then, development rate of urinary tract stones in the UK population stabilized. However, it still poses significant risk of mortality and morbidity in the elderly population [1]. The lifetime prevalence of urolithiasis, as reported in a 2016 study, based on hospital related admissions and intervention data, was 14%. [2].

A systematic review and meta-analysis undertaken by the NICE on management of ureteric stones (< 10 mm in size) identified that ureteroscopies are most effective in obtaining a stone-free status [4]. The guidelines further recommend offering surgical treatment to adult patients with ureteric stones within 48 h of diagnosis or of admission if the probability of the stone passing is remarkably low. The current surgical practice, however, has huge variations between the centres ranging from emergency treatment with primary intervention such as ureteroscopy, shock wave lithotripsy, percutaneous nephrolithotomy to symptomatic management with a temporary JJ ureteric stent insertion before undertaking scheduled definitive treatment. Safety and efficacy of emergency ureteroscopy have been reported in various studies [5–8], however, there exists uncertainty about whether treating the stone at initial presentation is more cost-effective over symptomatic temporizing stenting management followed by a definitive scheduled elective surgical procedure.

According to Getting It Right First Time (GIRFT) report in the UK [10], quality of care in acute stone disease in the UK contributes to significant morbidity and, therefore, needs further improvement. Similar to GIRFT findings, Scales et al. in a large study from United States involving 128, 564 emergency visits by patients with acute urolithiasis have clearly recommended further need for guidelines adherence and improvement. Failure to do so will increase variations in clinical practice and costs of care [9]. Reasons for not offering emergency ureteroscopic stone management in the UK, according to GIRFT report [10], were: lack of appropriate access to operating theatres, no nursing trained staff to assist in the use of laser, poor motivation to change practice and uncertain economic benefits.

With this background of scientific evidence, we made value-added changes to local pathways to reduce the time interval between CT detection of ureteric stones and their ureteroscopic laser fragmentation. The defining principle for these improvements was to ensure waste and duplication of care episodes were removed within the system and better outcomes are achieved at a reduced cost, similar to Lean method used elsewhere [11]. The purpose of this study was to assess clinical and cost effectiveness of the changes executed to shorten treatment episodes of obstructing stones by reducing the time interval between CT-detection and ureteroscopic laser-fragmentation. The approach to use time interval as marker of efficiency was modelled on the similar quality improvement project such as door to needle or door to balloon in other specialities [12]. The changes in pathway added value to patient journey and reduced clinical wastage. The study reports cost-effectiveness of quality improvement changes.

## Patients and methods

### Study cohort

The study cohort included all consecutive adult patients, who presented to two hospitals belonging to same health board, from a defined geographical area based around 75 primary care practices with CT-confirmed ureteric colic between 1st October 2018 and 31st December 2021 following implementation of the quality improvement clinical service.

The population of the region is registered with the healthcare facility through a single ten-digit community health index number (CHI number). The number is used as a unique identifier to carry out deterministic linkage of health records, investigations, scan results, operative notes, discharge documents, follow-up arrangements, clinic letters and clinical outcomes. The study had institutional approval (Caldicott Approval number #IGTCAL10483) and data can be made accessible to third party on request through institutional mechanisms.

The cohorts consisted of 100 patients who underwent Emergency Ureteroscopies with Laser fragmentation (EUL) and 20 patients who underwent ureteric JJ stent insertions followed by Delayed (elective) Ureteroscopies and Laser fragmentation (DUL) during the same study period. All procedures were carried out using same size ureteroscopes and disposable material with an average operative time of 40–50 min. This was a quality improvement project, hence power analysis and sample determination were not performed. Patients who had stents in place, had stents removed using flexible cystoscopy under local anaesthesia as an outpatient procedure. Table 1 below presents patient and stone characteristics of the cohorts under this study.

**Table 1:** Patient and stone characteristics

Characteristic*	EUL Emergency Ureteroscopy with Laser Fragmentation	DUL Delayed Ureteroscopy with Laser Fragmentation	*p* value
Number of patients	100	20	
Mean Age in years	55.66 (17.7)	66.73 (14.8)	0.011
Share of Males	73%	55%	0.048
Male: Female	1:2.7	1:1.2	
Mean Inpatient Duration in days	3.76 (7.14)	6.11 (5.20)	0.175
Mean Stone Size in millimetres	8.24 (4.46)	10.12 (4.72)	0.097
Share of proximal ureteric stones	33%	40%	0.550
Share of mid-ureteric stones	16%	35%	0.049
Share of distal ureteric stones	51%	25%	0.033

*Standard deviations in brackets. *p* < 0.05 is significant

### Exposure

We analyzed data of the study period following implementation of emergency ureteroscopy and laser fragmentation (EUL) facilities including training of staff. The change in pathways allowed us to offer quality improvement of clinical service. Study inclusion criteria were: non-pregnant patients, aged 19 years or older, who presented with acute ureteric colic secondary to CT-confirmed obstructing ureteric stone, where conservative treatment was thought to be less successful due to multiple factors like stone size (> 4 mm), degree of obstruction (presence of hydronephrosis) and symptoms (recurrent pain despite adequate analgesia). Primary treatment modality was set to be an emergency ureteroscopy with laser fragmentation (EUL). Only a small number of cases during the study period had urgent ureteric JJ stent insertion with delayed ureteroscopy with laser fragmentation (DUL). These patients underwent urgent stenting for symptom management. Patients with sepsis and those not fit for general anaesthesia underwent percutaneous placement of nephrostomy and delayed ureteroscopy. These patients were excluded and are not part of this study. DUL group was used for cost-analysis comparison. Patients who received other primary treatments or management modalities like shockwave lithotripsy or percutaneous nephrolithotomy on admission were excluded from this study. Patients who were awaiting elective ureteroscopies or have ongoing scheduled follow-up appointments with pending clinical outcomes, and recurrent stone formers were also excluded from this study.

### Outcomes

The primary outcomes of this study were single procedure stone-free rate (SFR) and cost-effectiveness. Stone clearance was defined as no residual stones at the time of procedure or on imaging at 3 months following index procedure (ureteroscopy and laser fragmentation). If the operating consultant was confident of no residual stones, the patients were deemed to have achieved stone clearance and no follow-up imaging was obtained.

Secondary outcomes were emergency re-admission to the hospital due to complications or an elective admission to the hospital for further management, and treatment, complications, and re-intervention rates. We define ‘re-admissions’ as any contact with the emergency services. However, some of these ‘re-admissions’ were due to minor stents related symptoms and did not involve any further surgical management.

### Costs

We retrieved all costs associated with interventions and common follow-up modalities from the NHS Cost of References 2019–2020 [13]. Tables 2, 3 below present all the cost information utilised for the purpose of our analysis—along with their NHS reference codes. Additionally, inpatient costs are retrieved from NHS National Statistics 2021 and account for £262 (€304) per bed per day [14]. The NHS unit cost data does not provide standard deviation information. We have added sensitivity analysis to the estimates accounting for ∓ 10% variation in both cost and probability data.

**Table 2:** Average costs associated with EUL patients in the quality improvement project with reference to the NHS Cost of References 2019–2020

EUL: Emergency Ureteroscopy with Laser fragmentation			
	Reference code	Reference descriptor	Cost (£)
Emergency ureteroscopy	LB65C–LB65E	Non-Elective Major Endoscopic, Kidney or Ureter Procedures, 19 years and over	£3,402.12
Stent removal	LB09D	Intermediate Ureter Procedures, 19 years and over	£171.42
Follow up imaging (USS)	RD40Z–RD42Z	Ultrasound Scan without contrast	£65.64
Others	RD23Z	Computerised Tomography Scan of Two Areas, without Contrast	£123.9
	PF	Plain Film	£33.61
	RN25A	Renogram, 19 years and older	£276.9
	LB36Z	Extracorporeal Lithotripsy	£331.2
	LB72A	Diagnostic Cystoscopy, 19 years and over	£171.38
	LB75A	Percutaneous Nephrolithotomy with CC score 2 +	£7,202.79
	LB75B	Percutaneous Nephrolithotomy with CC score 0–1	£2,546.12
	WF02C	Multi-professional Non-Admitted Non-Face-to-Face Attendance, Follow-up	£77.56

**Table 3:** Average costs associated with DUL patients in the quality improvement project with reference to the NHS Cost of References 2019–2020

DUL: emergency stenting followed by Delayed Ureteroscopies with Laser fragmentation			
	Reference Code	Reference Descriptor	Cost (£)
Emergency Stenting	LB09D	Non-Elective Intermediate Endoscopic Ureter Procedures, 19 years and over	£1,850.90
Elective Ureteroscopies	LB65C-LB65E	Elective Inpatient Major Endoscopic, Kidney or Ureter Procedures, 19 years and over	£2,854.44
Follow Up Stent Removal	LB09D	Intermediate Ureter Procedures, 19 years and over	£171.42
Follow Up Imaging (USS)	RD40Z-RD42Z	Ultrasound Scan without Contrast	£65.54
Others	RD23Z	Computerised Tomography Scan of Two Areas, without Contrast	£123.90
	PF	Plain Film	£33.61
	LB72A	Diagnostic Cystoscopy, 19 years and over	£171.38
	LB75A	Percutaneous Nephrolithotomy with CC score 2 +	£7,202.79
	LB75B	Percutaneous Nephrolithotomy with CC score 0–1	£2,546.12
	WF02C	Multi-professional Non-Admitted Non-Face-to-Face Attendance, Follow-up	£77.56

### Statistical analysis and cost effectiveness models

**Fig. 1:**
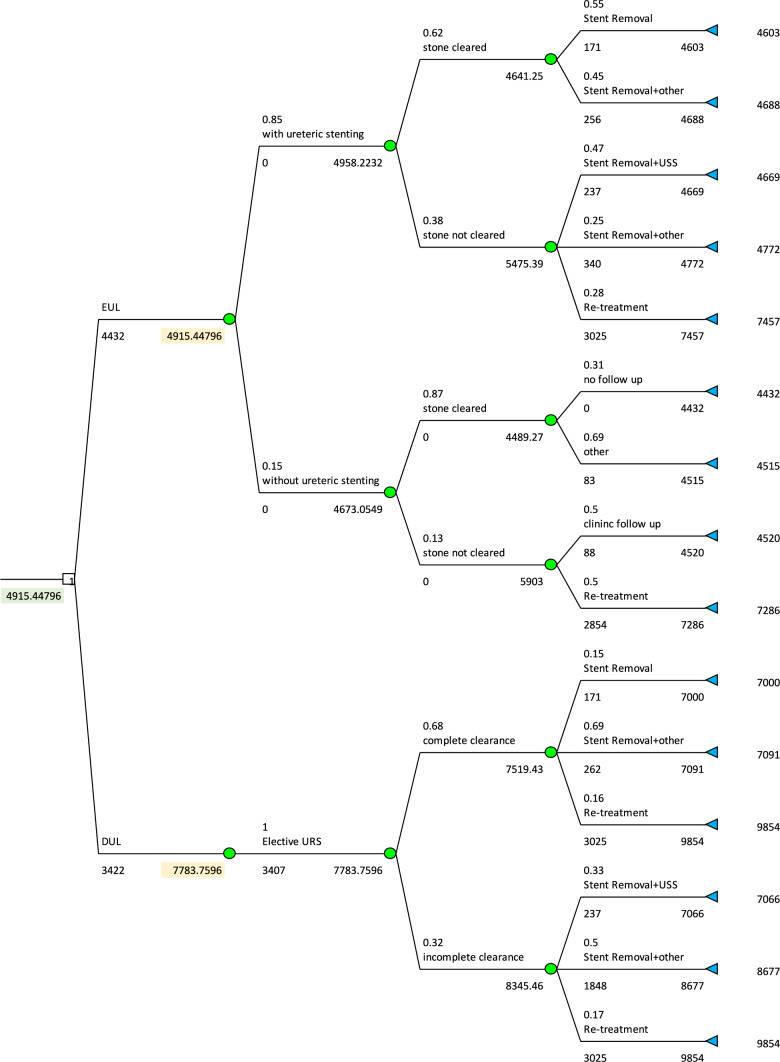
Decision Tree for cost-effectiveness analysis

There were numerous variables that were identified for the cohort of patients. Patients may or may not have stent insertions following emergency ureteroscopies with laser fragmentation which changes the course of follow-ups. Furthermore, following initial management with aforementioned treatment options, patients had different follow-up modalities to check primary outcome, some of which included follow-up stent removals, ultrasound scans, CT scans, and x-rays. Therefore, a diagram was formulated based on the clinical decisions undertaken during the clinical episodes to allow a better understanding of different variables involved in the study (Figure A-supplementary). This diagram was then used to develop a decision tree for our cost-effectiveness analysis. This decision tree is developed using TreePlan Software^©^ and is illustrated in Fig. 1. As shown, the tree initiates with two branches, EUL and DUL. Each of the branches are then guided by the sequence of event nodes, including initial admission, procedure, and outcome of the treatment. In case the patient was re-admitted, the cost of the re-admission process is also included using observed cost and probability data of our cohorts. Therefore, our analysis includes both—the initial visit and any re-admission treatments. Based on the numerous variables and branches, a cost-analysis was performed to ascertain and identify the more cost-effective treatment modality for managing adult patients with ureteric stones. The latest NHS Cost of References 2019–2020 was used to calculate the costs of procedures and events which are part of the study. Average cost was calculated and used for the different length of hospital admissions and tests to allow for a fair and accurate cost analysis. A brief cost-effectiveness assessment was also included as part of this study by analysing the real cost of in-patient days after excluding cost of hospital beds from cost calculations. Statistical analyses are conducted using Stata/MP 17.

## Results

Single procedure stone-free rate at 3 months for EUL and DUL cohorts was 85% for both. Therefore, there were no significant differences in clinical effectiveness. Average (standard deviation) time between CT-detection and laser fragmentation for EUL was 2.13 days (± 5.7) vs. for DUL was 91.63 days (± 50.6). Re-admission rate for EUL and DUL patients was 18% and 20% respectively (see Table 4). Most common reason for re-admissions was stent-related symptoms. In many centres and regions this is considered as re-attendance and not re-admission. However, in our centre, electronic systems in the healthcare board showed these as ‘admissions’, and hence have been noted under the respective category for this study. The intra-/post-operative complication rate was 4% in the EUL cohort vs 5% in the DUL cohort. These included three urinary tract infections (Clavien–Dindo class II), 1 dislodged stent requiring removal under general anaesthesia (Clavien–Dindo class III) in the EUL group, and one stent encrustation requiring ureteroscopic laser fragmentation (Clavien–Dindo class III) in the DUL group.

**Table 4:** Secondary outcomes results for EUL and DUL

Outcomes	Total	EUL	DUL	*p* value
Complications	4.1% *(2%)*	4% *(19%) [4/100]*	5% *(22%) [1/20]*	0.839
Complete clearance	66.6% *(47%)*	66% *(47%) [66/100]*	70% *(47%) [14/20]*	0.731
Re-intervention	10% (30%)	9% (28%) [9/100]	15% (36%) [3/20]	0.418
Re-admission	18.3 *(46%)*	18% *(38%) [18/100]*	20% *(41%) [4/20]*	0.834

^*^Standard deviations in round brackets. Number of occurrences over total sample in square brackets

Stone clearance was defined as no residual stones on imaging at 3 months following index procedure (ureteroscopy and laser fragmentation). Almost all patients in the DUL ureteroscopy cohort (18 out of 20 patients – 90%) required subsequent outpatient radiological investigations such as a CT Kidneys, Ureters, and Bladder, or an Ultrasound scan of the urinary tract to ensure complete stone clearance. Whereas only 30 patients (30%) in EUL group required follow-up US or CT scans. If operating physician was confident about leaving no residual fragments after ureteroscopic laser fragmentation, no follow-up imaging was requested. If these patients had no re-admissions at 3 months, they were deemed to have achieved stone clearance.

Cost for implementing the quality improvement project (reduction in CT detection to surgical intervention with emergency ureteroscopy and laser fragmentation—EUL) amounted to £4915 (€5580) per patient, while costs for delayed ureteroscopy and laser fragmentation (DUL) was accounted to be £7783 (€8847) per patient. The estimated cost savings per averted delayed ureteroscopy was £2868 (€3260) per patient (see Table 5).

**Table 5:** Cost-effectiveness analysis with respect to inpatient days

	Cost (£)*	In-patient days	ICER (£)
DUL	7783 [6982, 8578]^1^	6.11	–
EUL	4915 [4403, 5398]^1^	3.76	1220 [674, 1776]^1^

^1^Min and max interval based on varying cost and probability data within ∓ 10% range

## Discussion

Several studies in different areas have reported a significant quality improvement by reducing time to delivery of treatment after reporting to hospitals as surrogate marker of quality in the healthcare system [15–17]. Similar exemplar reports in urological clinical practice, however, do not exist. Stone disease is a common and painful condition associated with significant morbidity in most productive years of life and, therefore, warrant reports focusing on time to delivery of treatment [18].

Management of urological stone disease is built on a complex network of care processes and pathways. The efficiency and quality of care delivered depend on how well resources could be managed and duplications and waste could be avoided. For example, tackling variations in clinical practice or activities between providers can significantly improve quality of care. The basic steps of any improvement project are: understanding the challenge, designing and implanting improvement, data collection, and measurement of improvement, sustainability, and effective communication with all the stakeholders.

There are various methods of quality improvement, and many have come to healthcare through experience in industry. Planning, doing, studying and acting (PDSA) are simplest forms of quality improvement approach which runs in small cycles [19]. Lean method popularized by Japanese is centred on patient value flow and involves cutting down anything which does not benefit patients [11, 20]. Co-designing quality improvement system in consultation with patients has been practised in the UK [21]. There is a great need for quality improvement in stone disease management as this a benign disease with known recurrences and focus on achieving stone-free status as soon as possible [22].

Getting It Right First Time (GIRFT)—a National Health Service (NHS) quality improvement programme aimed at reducing unwarranted variation has identified acute stone presentation as one the improvement projects. This is an example of a large scale response to quality improvement in the healthcare backed by the UK government to improve productivity in the NHS following Lord Carter’s report in 2016 [23]. GIRFT in urology [10] identified that patients who undergo temporary stenting for acute urinary obstruction are not provided opportunity of one-stop, definitive stone treatment and suffer from long-wait, re-admissions with stent related pain resulting in poorer quality of care. Access to acute definitive treatment with Extra-corporeal Shock Wave Lithotripsy (ESWL) was found to be poor as well. We established acute stone service for patients in our regions catering a population of more than 4,170,000 [24, 25] with the intention of addressing concerns raised by GIRFT report in urology.

Acute stone presentation with urinary obstruction or symptoms can be managed by a period of conservative treatment with medical expulsive therapy, stenting alone, ureteroscopy and laser fragmentation or ESWL. Ureteroscopic laser fragmentation or intact stone extraction has been found to be most effective with high stone-free rates and its clinical safety in emergency has been confirmed [26]. The single procedure stone-free rates range from 81 to 100% and have been reported in numerous studies analysing ureteroscopies [26–28]. Anagnostou et al. recommend managing large ureteric stones with initial laser ureteroscopies, given its overall better outcome in comparison to other modes of management such as ESWL [28].

It was, therefore, hypothesized prior to instatement of the quality improvement project at our centre that emergency ureteroscopies with laser fragmentation (EUL) would not only reduce the time interval of achieving a stone-free status but also deliver a stone-free status in a clinical and cost effective manner without compromising patient safety. With an equal 85% single procedure stone-free rate at 3 months, our cohorts shared similar clinical effectiveness with marginally improved secondary outcomes in the EUL group. The most significant difference noted between both the cohorts was the time interval between CT-detection and ureteroscopy with laser fragmentation—average of 2.13 days and 91.63 days in EUL and DUL groups respectively. Additionally, there is a statistically significant age difference between groups in this study, and gender balance is marginally unbalanced. While the overall difference between stone sizes is not statistically significant, there are some size differences in distal ureteric stones.

Numerous other studies have evaluated the clinical effectiveness of emergency ureteroscopies and had similar outcomes to delayed ureteroscopic management with same associated morbidity but decrease in waiting time. These have been summarized in Table A (supplementary). In comparison to other modalities of managing acute stone episodes, emergency ureteroscopic management of ureteric stones has been reported to be superior by numerous studies [26–28]. All the studies were able to establish > 80% stone-free rates, low morbidity, and excellent patient recovery. Our findings confirm clinical effectiveness of emergency ureteroscopic laser fragmentation like previously reported outcomes in a different population.

Cost effectiveness of emergency ureteroscopic laser fragmentation using decision tree analysis or Markov’s model has not been reported. A retrospective cost analysis performed by Youn et al. [29] showed that the mean hospital cost for patients who underwent emergency ureteroscopies was KRW525,350 (GBP 335.69/€390) vs. KRW 762,360 (GBP 487.14/€566 for patients who underwent delayed ureteroscopies, concluding that total cost per patient was less when ureteroscopies was performed emergently. The cost-effectiveness of emergency ureteroscopic approach reported in this Korean study confirms our findings and validates the assumption that the approach of achieving an early stone-free status can financially benefit healthcare system outside the UK.

In clinical service at our centres, EUL were found to be more cost-effective saving approximately £2868 per patient in our study. The additional cost associated with the DUL cohort may be attributed to increased number of surgical procedures, follow-up and imaging modalities, overall inpatient stay, and readmissions. All patients in the DUL cohort were noted to have at least two hospital admissions (1st admission for emergency stenting and a subsequent elective admission for ureteroscopic fragmentation) thereby factoring additional costs towards management of ureteric stones in this group. Cost saving as observed by us is similar to reported by Wani et al. [30], however, detailed cost analysis using discrete time decision tree model was not used in the later. Decision trees are economic models commonly used in healthcare to calculate spend of money associated with follow-up decisions and chance episodes overtime, including complications and re-admissions. The paradigm of model—first developed by the Russian scientist Andrei Markov (1856–1922) is based on partially cyclic directed graphs comprises: the decision node, the decision strategy, and the outcome nodes [31, 32]. Figure 1 in our study shows analytic model with attached costs and weights to the health states during emergency ureteroscopic laser fragmentation and follow-up health outcomes. Based on the model, a cost-effective proposal for the future service design could be formulated with offer of ureteroscopic laser fragmentation as outpatient procedure without stent placement. That would also avoid 2 days hospital stay observed in this study between CT-detection of stone and ureteroscopy.

Our study is limited by a lack of comparative group in its design as well as the significant size difference between the study cohort and the comparison cohort. The 91-day wait prior to treatment in the delayed group can be considered as too long and certainly has potential to increase complication rate and re-admission. Our study reflects real clinical practice data. The delays in delivery of treatment during the study period may also be attributed to the impacts of COVID-19 pandemic. Another acknowledged source of bias in the present study is the difference in stone location between the groups. There was a significant difference in distal ureteric stones between Emergency and delayed ureteroscopy groups (51% vs 25%; *p* = 0.033, in emergency and delayed group respectively). Our stone-free definition may be a limitation, however there is no universally agreed definition, and many, similar to us would believe endoscopic assessment is acceptable (33).

The study does, however, provide reasonable strong evidence in its cost analysis in the absence of local and international guidelines regarding the preference of ureteroscopies in an emergency setting. The evidence produced through this study could be used as case support for quality improvement approach in designing acute stone service with significant benefits to patients (clinical effectiveness) and cost-saving to healthcare systems. The study has shown improvement in productivity and financial cost, however admittedly we do not have patient-reported outcomes and an important ingredient of data on user-experience is lacking. Although, clinically we assume a better quality of life in those who are stone free, quality of life data and its incorporation into model is a limitation in the present study. In the future, we suggest more data coming from other centres with robust quality assurance and quality of life data to make quality improvement as core-business in acute urology stone management.

## Conclusion

Emergency ureteroscopies and laser fragmentation of stones as a quality improvement project based on reduction in CT detection-to-laser fragmentation time is both clinically and economically effective. The presented cost-effectiveness data provide evidence to guide healthcare leaders planning similar interventions at other centres.

## Supplementary Information

Below is the link to the electronic supplementary material.

Supplementary file1 (DOCX 180 KB)

## Data Availability

The data can be shared on request and using our institutional policy on data sharing.
