# Supplementary material for: Cost-effectiveness of quality improvement intervention to reduce time between CT-detection and ureteroscopic laser fragmentation in acute symptomatic ureteric stones management
Source: World J Urol. 2024 Mar 13;42(1):144. doi: 10.1007/s00345-023-04694-4 (PMC10937764; doi:10.1007/s00345-023-04694-4)
Supplement: Supplementary file 1 — Supplementary file1 (DOCX 180 KB) [file 345_2023_4694_MOESM1_ESM.docx]

**SUPPLEMENTARY ANNEXE**


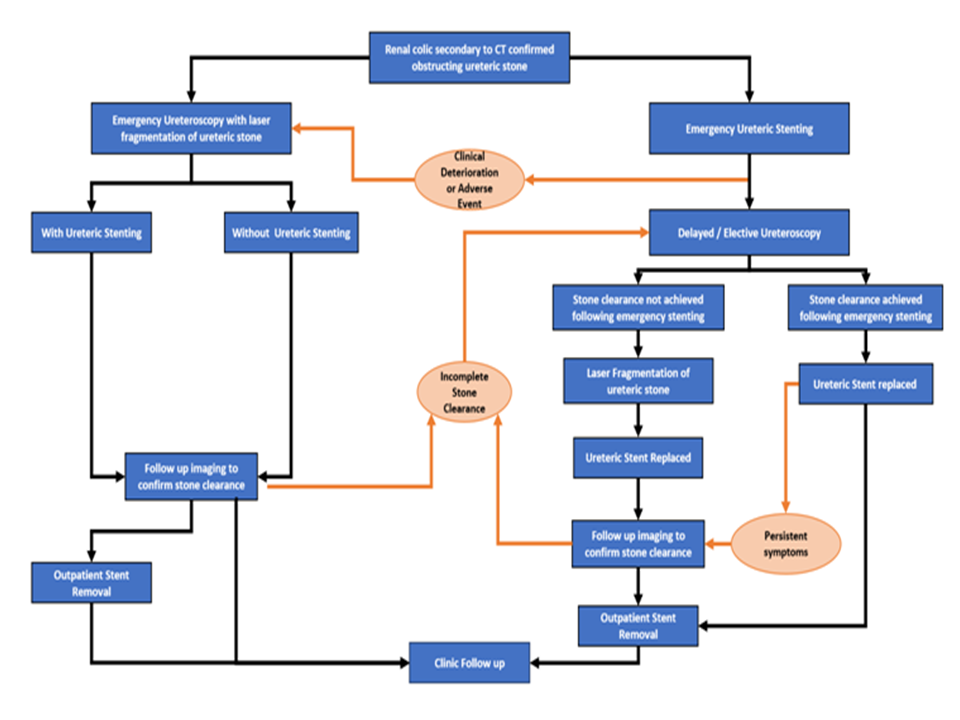


*Figure A: Summary and flow of the study*

| Study (authors) | Khopekar et al. | Al-Ghazo et al.^[12]^ | Osorio et al.^[13]^ | Yang et al.^[14]^ | Youn et al.^[15]^ |
| --- | --- | --- | --- | --- | --- |
| Patients (numbers) | 100 | 244 | 144 | 49 | 67 |
| Age (years) | 55.6 | 45.6 | 49.6 | NA | 47.8 |
| Average Size | 8.24mm | 9.2mm | 9.1mm | 14.8mm | 2.41mm |
| Stone-Free Rate (%) | 85 | 90.6 | 92.4 | 91.8 | 89.55 |
| Follow-up | 3 months | 4 weeks | 1 month | 3 months | 1 month |
| Hospital Stay | 3.8 days | 1.9 days | 2.5 days | NA | NA |

*Table A: Summary of studies reporting emergency ureteroscopies analyses comparison*

| Study (authors) | Khopekar et al. | Al-Ghazo et al.^[12]^ | Osorio et al.^[13]^ | Yang et al.^[14]^ | Youn et al.^[15]^ |
| --- | --- | --- | --- | --- | --- |
| Patients (numbers) | 100 | 244 | 144 | 49 | 67 |
| Age (years) | 55.6 | 45.6 | 49.6 | NA | 47.8 |
| Average Size | 8.24mm | 9.2mm | 9.1mm | 14.8mm | 2.41mm |
| Stone-Free Rate (%) | 85 | 90.6 | 92.4 | 91.8 | 89.55 |
| Follow-up | 3 months | 4 weeks | 1 month | 3 months | 1 month |
| Hospital Stay | 3.8 days | 1.9 days | 2.5 days | NA | NA |
